# New Implications of Metabolites and Free Fatty Acids in Quality Control of Crossbred Wagyu Beef during Wet Aging Cold Storage

**DOI:** 10.3390/metabo14020095

**Published:** 2024-01-29

**Authors:** Shuji Ueda, Yuka Yoshida, Biniam Kebede, Chiaki Kitamura, Ryo Sasaki, Masakazu Shinohara, Itsuko Fukuda, Yasuhito Shirai

**Affiliations:** 1 Department of Agrobioscience, Graduate School of Agricultural Science, Kobe University, Hyogo 657-8501, Japan; 236a414a@stu.kobe-u.ac.jp (C.K.); itsuko@silver.kobe-u.ac.jp (I.F.); shirai@kobe-u.ac.jp (Y.S.); 2 Japan Meat Science and Technology Institute, Tokyo 150-0013, Japan; y.yoshida@niku-kakou.or.jp; 3 Department of Food Science, University of Otago, P.O. Box 56, Dunedin 9054, New Zealand; biniam.kebede@otago.ac.nz; 4 Food Oil and Fat Research Laboratory, Miyoshi Oil & Fat Co., Ltd., Tokyo 124-8510, Japan; sasakir@miyoshi-yushi.co.jp; 5 The Integrated Center for Mass Spectrometry, Kobe University Graduate School of Medicine, Hyogo 650-0017, Japan; mashino@med.kobe-u.ac.jp

**Keywords:** Wagyu, metabolomics, foodomics, triacylglyceride, cold storage, free fatty acids, umami, kokumi, food loss

## Abstract

Efficient cold-chain delivery is essential for maintaining a sustainable global food supply. This study used metabolomic analysis to examine meat quality changes during the “wet aging” of crossbred Wagyu beef during cold storage. The longissimus thoracic (Loin) and adductor muscles (Round) of hybrid Wagyu beef, a cross between the Japanese Black and Holstein–Friesian breeds, were packaged in vacuum film and refrigerated for up to 40 days. Sensory evaluation indicated an increase in the umami and kokumi taste owing to wet aging. Comprehensive analysis using gas chromatography-mass spectrometry identified metabolite changes during wet aging. In the Loin, 94 metabolites increased, and 24 decreased; in the Round, 91 increased and 18 decreased. Metabolites contributing to the umami taste of the meat showed different profiles during wet aging. Glutamic acid increased in a cold storage-dependent manner, whereas creatinine and inosinic acid degraded rapidly even during cold storage. In terms of lipids, wet aging led to an increase in free fatty acids. In particular, linoleic acid, a polyunsaturated fatty acid, increased significantly among the free fatty acids. These results provide new insight into the effects of wet aging on Wagyu-type beef, emphasizing the role of free amino acids, organic acids, and free fatty acids generated during cold storage.

## 1. Introduction

Global meat consumption has consistently increased. Annual consumption is expected to reach 50 kg/person by 2030 [1]. One notable livestock breed in Japan is the Japanese Black cattle, commonly called Japanese Wagyu. This breed has evolved as a native animal and adapted to the Japanese climate and agricultural environment. Japanese Wagyu beef is characterized by excellent marbling from intramuscular fat and a sweet aroma which is produced when cooked [2,3]. Marbling is a unique genetic trait of Japanese Wagyu cattle, and the intramuscular fat-related genes have attracted interest in the research community [4]. Marbling is one of the critical factors that determine the value of beef to consumers. Hybrid Wagyu, a crossbreed of Japanese Wagyu with other breeds, is used to produce marbled meat efficiently.

Japanese Wagyu are raised on controlled grain feed in cattle barns and are typically sent for slaughter at 28 months or older [5]. In Japan, hybrid Wagyu is commonly bred through the artificial insemination of the Holstein–Friesian breed with Japanese Wagyu semen. Notably, hybrid Wagyu exhibits lower marbling content compared to its purebred Japanese Wagyu counterpart [6,7] but has the advantage of a shorter fattening period (about 26 months of age) owing to the genetic influence of the Holstein breed [8].

A new approach to improving the efficiency of livestock production focuses on improving logistics efficiency [9]. An efficient meat supply chain from the meat center to the consumer is expected to reduce food loss and waste [10]. High-quality beef is film vacuum-packed and refrigerated to avoid deterioration in meat quality during delivery and storage [11]. In cold storage, nutrients in the meat are broken down into free amino acids, monosaccharides, and peptides by enzyme activity which remains in the muscle after slaughter. The favorable changes in meat quality during cold storage are referred to as wet aging, and the metabolites that increase during aging act as substrates for the Maillard reaction that occurs during cooking, contributing to the formation of the beef’s characteristic flavor [12]. 

Metabolomics, in which mass spectrometry is employed to comprehensively examine metabolite profiles, has markedly advanced our understanding of metabolites which are closely associated with meat aroma, texture, marbling, and color [13,14]. Specifically, metabolomics analysis enables a thorough exploration of metabolites and offers valuable insights into changes in meat quality during the postmortem aging process [15,16]. 

However, metabolomics analysis in crossbred cattle remains limited, primarily as Japan has traditionally emphasized research on purebred Wagyu cattle. Herein, we employed metabolomics to investigate metabolites and lipids during the wet aging of beef packaged in an oxygen barrier film, focusing on hybrid Wagyu cattle rather than purebred Japanese Black cattle. We reveal temporal changes in metabolites and free fatty acids (FFAs) associated with meat quality during cold storage, providing information on metabolic markers that could enhance quality control measures.

## 2. Materials and Methods

### 2.1. Sample Collection and Cold Storage

Blocks of the Loin (longissimus thoracic muscle) and Round (adductor muscle) of crossbred cattle, a hybrid of Japanese Black and Holstein–Friesian breeds, were procured commercially from livestock farmers in Hokkaido, Japan. We used the Loin and Round from five crossbred cows for this study. The right side of each portion was used for metabolomics analysis, whereas the left side was used for sensory evaluation and analysis of each component. All cows were 26-month-old castrated cows with a marbling score of 3, as per the beef marbling standard defined by the Japan Meat Grading Association. For cold storage, the Loin and Round muscles were subdivided into five equal portions at the meat processing center and vacuum-packed with oxygen barrier film (Barrialon S, Asahi Kasei Co., Tokyo, Japan). On Day 0, samples were frozen immediately after packaging. The remaining samples were incubated in a refrigerator (temperature setting: 2 °C, 75% humidity) for 10, 20, 30, and 40 days of cold storage, and then frozen at −30 °C. Details of the packaging films are listed in Table 1. This study did not include any animal experiments.

### 2.2. Sensory Evaluation

A sensory panel comprising five members with experience in analyzing meat samples evaluated the taste profile of the crossbred Wagyu beef. The panelists comprised experts with long-term practical experience in sensory evaluation of meat. A barbecue-cut sample (40 mm × 50 mm, thickness: 10 mm) was placed in a vat and exposed to air overnight in a refrigerator at 4 °C. The sample was cooked as a grilled steak by heating at 230 °C for 60 s and then cooking the backside for 90 s with an electric griddle (Zojirushi, Tokyo, Japan). In compliance with the sensory evaluation guidelines for meat (National Livestock Breeding Center, Tokyo, Japan), cooked beef was served as a blind sample, one at a time, in the sensory evaluation room (2–3 samples per day). Sensory evaluation was performed for seven parameters (salty, sweet, sour, bitter, umami, richness, and continuity), with the evaluation scale ranging from 0.00 to 2.00. The rating scale for cold storage Day 0 beef was set at 0.00 as a standard.

### 2.3. Metabolomics Analysis by GC/MS

Frozen beef samples (1 g) were finely shredded and crushed in a multi-bead shocker (μT-48, Tokken Inc., Chiba, Japan) fitted with a stainless-steel holder (TK-AM7-H, Tokken). One mL of solvent (1 mL; methanol: chloroform: water, 2.5:1:1) containing an internal standard (5 mg of sinapic acid) was added to the crushed beef sample (10 mg) and stirred using a rotator (1500 rpm, 5 min). The homogenate was centrifuged at 15,000× *g* for 5 min, and the supernatant was collected as a water-soluble extract. 

The water-soluble extract was completely dried using a centrifugal evaporator (Tokyo Rikakikai Co., Tokyo, Japan) for 60 min and freeze-dried (Tokyo Rikakikai) for 16 h. The dried samples were dissolved in 80 μL of pyridine containing 20 mg/mL methoxyamine hydrochloride (Sigma-Aldrich Japan KK, Tokyo, Japan) for 30 min while shaking at 1200 rpm. The mixture was derivatized with 40 μL of N-methyl-N-trimethylsilyltrifluoroacetamide (GL Sciences Inc., Tokyo, Japan) for 30 min with shaking at 1200 rpm at 37 °C. After centrifugation at 16,000× *g* for 5 min at 4 °C, 50 μL of the resulting supernatant was analyzed by GC/MS.

GC/MS analysis was performed on a GCMS–QP2010 Ultra (Shimadzu Co., Kyoto, Japan) with a DB-5 capillary column (30 m × 0.25 mm; film thickness, 1.0 µm; Agilent Technologies, Santa Clara, CA, USA). The mass spectrum was analyzed using the GC/MS Metabolite Database v.2 (Shimadzu). Detailed analytical conditions were the same as in a previous study [16].

### 2.4. Analysis of the Triacylglycerides, Free Amino Acids, and Nucleic Acid-Related Components

Samples dissolved in isopropyl alcohol were analyzed by high-performance liquid chromatography using Agilent Technologies 1260 Infinity equipped with a refractive index detector and Poroshell 120 EC-C18 LC column (three columns in a series: 3.0 mm × 50 mm, 3.0 mm × 50 mm, and 3.0 mm × 100 mm; 2.7-Micron; Agilent Technologies) following a previously described method. Individual TG species were quantified by determining the corresponding relative percentage according to the normalization area procedure [14]. 

Free amino acid analysis was performed using the conventional post-label ninhydrin method using column derivatization high-performance liquid chromatography (L-8900 type high-performance amino acid analyzer; Hitachi High-Tech Corporation, Tokyo, Japan) with a strongly acidic cation exchange resin-packed column [17].

Analysis of nucleic acid-related metabolites was performed as described in a previous study by Ichimura et al. Quantification of nucleic acid-related substances was analyzed by high-performance liquid chromatography using a Shimpack HRC-ODS column (6.0 mm inner diameter × 150 mm; Shimadzu Co.).

### 2.5. Analysis of Total Fatty Acid and Free Fatty Acids

For total lipids, 5 g of finely ground beef was extracted with an organic solvent (methanol: chloroform = 1:1) according to the Folch method, and the lipid fraction was collected. Heneicosanoic acid (C21:0) was added as an internal standard, and FFAs were collected using a solid phase column (InertSep SI; GL Sciences). The solvent was removed by drying under nitrogen. For FFAs, the lipid fraction extracted by the Folch method was subjected to a solid phase extraction column (Bond Elut NH2; Agilent, Santa Clara, CA, USA), and the FFA fraction was recovered [14]. 

The total fatty acid and FFAs were methyl esterified with boron trifluoride methanol solution. Samples were collected with saturated sodium chloride n-hexane, dehydrated with anhydrous sodium sulfate, and subjected to gas chromatography with FID (GC-2010 plus, Shimadzu). The separation column was an SP-2560 column (0.25 mm, length 100 m, film pressure 0.20 μm, Supelco Inc., Bellefonte, PA, USA). Analytical conditions were as follows: inlet temperature: 250 °C; detector temperature: 250 °C; column temperature program: 180 °C (150 min) gradient (10 °C/min) and 220 °C (10 min) [14].

### 2.6. Statistical Analyses

Multivariate analysis (PCA) was performed using SIMCA 14.1 software (Infocom, Tokyo, Japan). Heatmaps were drawn using Heatmapper (http://www.heatmapper.ca/, accessed on 24 December 2022) [18]. Statistical significance was determined using a Student’s *t*-test by Excel 2019 (Microsoft Japan, Tokyo, Japan), or a Tukey–Kramer method bellcurve (Social Survey Research Information, Tokyo, Japan).

## 3. Results

### 3.1. Changes in the Taste of Hybrid Wagyu Beef during Wet Aging

**Figure 1:**
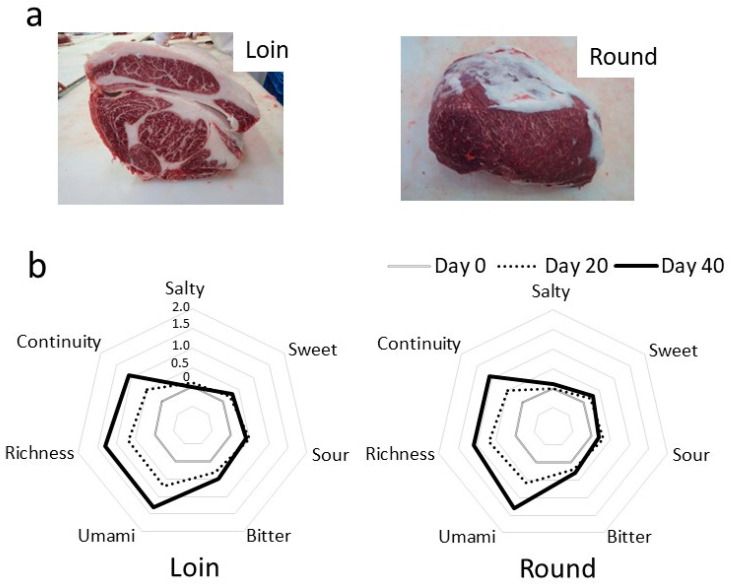
Sensory evaluation of hybrid Wagyu beef after cold storage. (**a**) Photographs show hybrid Wagyu beef’s longissimus thoracis (Loin) and adductor muscle (Round) cuts. (**b**) Radar chart showing the strength of each taste. The roasted beef was sensory evaluated by expert panelists (values are means of five cattle conducted by five panelists). The strength of each item was compared on Day 20 (dashed line) and Day 40 (bold line) relative to Day 0 (gray line; score set at 0) as the standard.

To assess changes in the quality of hybrid Wagyu (Japanese Black and Holstein–Friesian breeds) beef during wet aging in a refrigerator, blocks (Day 4 after slaughter) of longissimus thoracis (Loin) and adductor muscle (Round) were packaged with oxygen barrier film and stored in a refrigerator for 0–40 days. Similar to previous studies, changes in taste due to wet aging were evaluated in a sensory evaluation test conducted by expert panelists [16]. Sensory evaluation was performed by comparing beef on Day 20 and Day 40 after cold storage, with Day 0 of storage being the reference. Sensory evaluation scores are presented in a radar chart (Figure 1).

Scores for umami, richness, and continuity (a sustained boiled-meat-like flavor) increased for both the Loin and Round as storage duration progressed from Day 20 to Day 40. Conversely, scores for saltiness, sweetness, and acidity exhibited minimal change over storage time. Notably, even after 40 days, neither oxidized nor foul odors (such as metal or acid aromas) were detected in the Loin and Round. These sensory evaluation results align with previous reports of taste changes due to cold storage [19].

### 3.2. Comprehensive GC/MS Analysis of Metabolite Changes during Wet Aging

**Figure 2:**
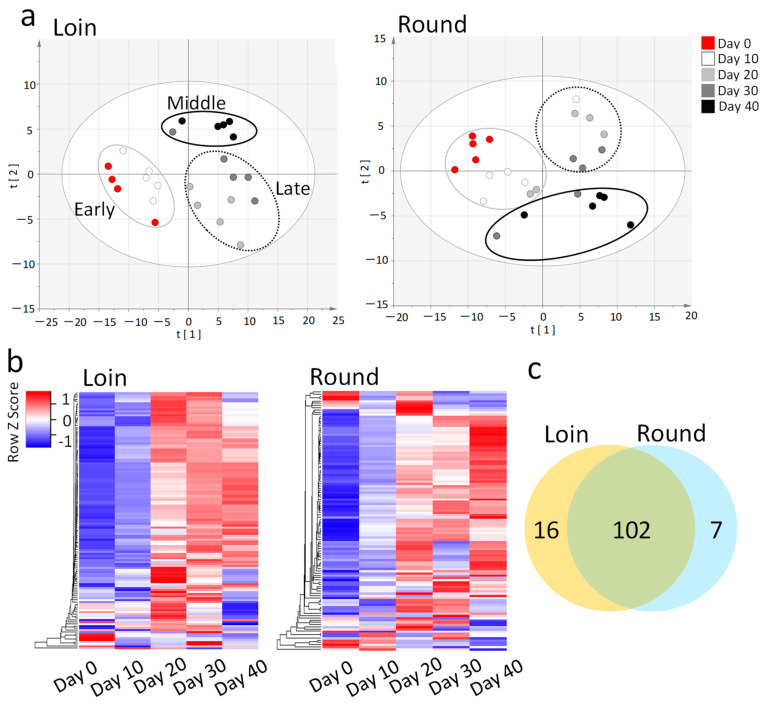
Metabolomics analysis of hybrid Wagyu beef during cold storage. (**a**) The score plots of principal component analysis (PCA) of the metabolites. Vacuum-packed longissimus thoracis (Loin) and adductor muscle (Round) cuts of hybrid Wagyu beef were refrigerated from Day 0 to Day 40. The water-soluble fraction of the sample was prepared and analyzed by gas chromatography–mass spectrometry (each storage time for five cattle). The PCA score plot classified the Loin and Round sample groups into three groups according to metabolites: early (gray line), middle (dotted line), and late (solid line). The PCA score plots show Loins (R2X (1) = 0.488; R2X (2) = 0.116) and Rounds (R2X (1) = 0.440; R2X (2) = 0.135). Scaling of PCA was performed in UV mode. One sample was missing on Day 0 for Loin because of machine trouble. (**b**) Heat map obtained from hierarchical cluster analysis. The heat map visually shows changes in metabolites associated with cold storage. Horizontal columns in the heat map represent metabolites, and vertical columns represent cold storage time. Blue indicates low concentrations and red indicates high concentrations of metabolites. The color gradients in the heat map indicate changes in metabolite concentrations with cold storage time. Cluster analysis of heat map is performed using the clustering method (single linkage) and distance measurement (Pearson). (**c**) The Venn diagram shows the number of overlapping metabolites in the Loin and Round.

Since various metabolites such as amino acids, organic acids, nucleotides, and carbohydrates act synergistically on beef taste and overall flavor, the profile of these metabolites is closely related to meat quality [20]. We used gas chromatography–mass spectrometry (GC/MS) to examine the metabolites that changed in the hybrid Wagyu beef during cold storage. The metabolomics analysis identified many metabolites in the Loin (118 metabolites) and Round (109 metabolites). Principal component analysis (PCA) was conducted as a preliminary, unsupervised, explorative analysis on the metabolite data. The PCA score plots classified the analyzed samples into three groups (early, middle, and late) based on the metabolites (Figure 2a). 

A heat map image illustrates the correlation between cold storage time and metabolite concentration (Figure 2b). The shading in the map clearly shows the patterns of increase and decrease in the metabolites during cold storage. Of the detected metabolites, 102 were present in the Loin and Round (Figure 2c). Among the metabolites, 2,3-bisphosphate-glyceric acid, glutaric acid, citrulline, maleic acid, and ethylmalonic acid were exclusively detected in the Loin, while trehalose, mannose, xylitol, and nicotinic acid were found only in the Round.

**Figure 3:**
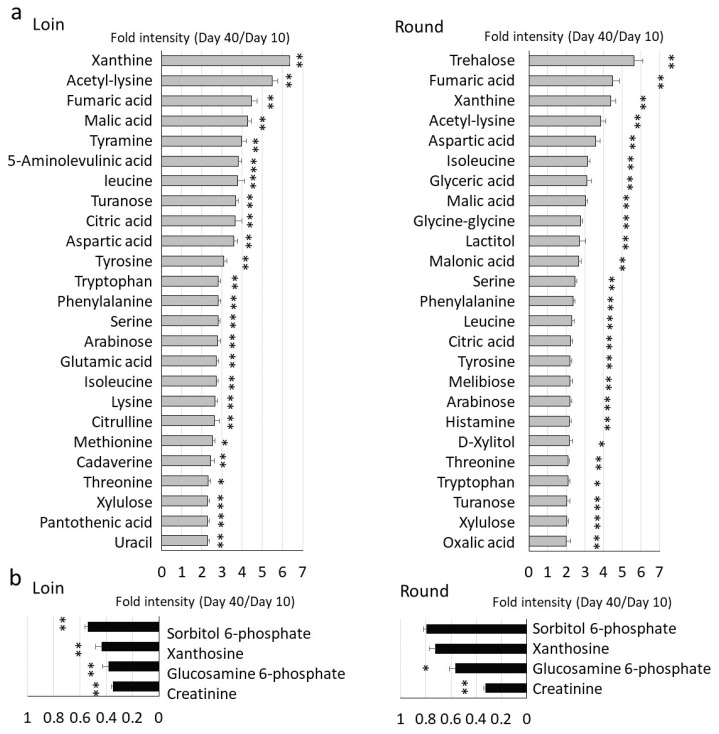
Metabolites significantly increased or decreased with cold storage. (**a**) Fold-change in the top 25 increased metabolites with cold storage. The left graphs show longissimus thoracis (Loin), and the right graphs show adductor muscles (Round). (**b**) Fold-change in decreased metabolites with cold storage. Values in the graph are means of relative values at Day 40. Graphs show metabolites that showed significant differences (Student’s *t*-test; *n* = 5; ** *p* < 0.05, * *p* < 0.05). Error bars mean ± SD.

Most metabolites increased with cold storage, reaching their peak on Days 30 or 40 (Figure 2b). The top 25 metabolites that showed significant increases (Day 10 vs. Day 40) are shown in the graph (Figure 3a). Amino acids (such as leucine, tyrosine, serine, and isoleucine) and tricarboxylic acid metabolites (fumaric acid, maleic acid, and citric acid), which are associated with the citric acid cycle, dominated the metabolites that significantly increased during cold storage [21]. Additionally, glycolytic metabolites (turanose, xylulose, and trehalose) showed increased levels during cold storage. By contrast, creatinine, glucosamine 6-phosphate, xanthosine, and sorbitol 6-phosphate levels decreased during cold storage (Figure 3b). 

**Figure 4:**
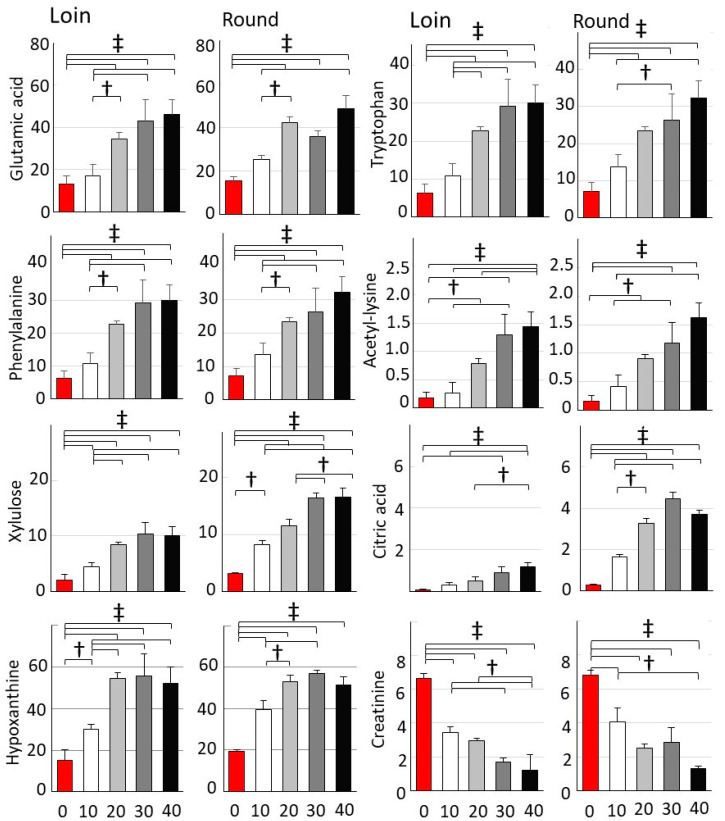
Temporal changes in metabolites during cold storage. Metabolites exhibiting increased and decreased levels during cold storage. Graphs show the mean values of metabolites and metabolites exhibiting significant differences based on cold storage duration (Tukey–Kramer; *n* = 5; † *p* < 0.05, ‡ *p* < 0.05). Error bars mean ± SD.

The graph depicts the characteristic metabolites identified in the GC/MS analysis (Figure 4). Glutamic acid contributes to the umami taste, whereas tryptophan, phenylalanine, and hypoxanthine are associated with bitterness, and creatinine enhances taste continuity [22,23]. The levels of these metabolites showed substantial increases or decreases over time. These metabolites may serve as biological markers for assessing taste alterations in hybrid Wagyu beef owing to wet aging. Among the detected metabolites, acetylated lysine, a posttranslationallymodified amino acid, showed an increase over time [24].

Nucleic acid-related metabolites play a crucial role in shaping the taste of beef [25]. To quantify nucleic acid-related metabolites, we used high-performance liquid chromatography alongside metabolomics analysis (Figure S1). Among the metabolites examined, inosinic acid, a key contributor to umami, exhibited rapidly decreased levels during cold storage. 

### 3.3. Comparison of Lipid Composition in Hybrid Wagyu Beef

Herein, we investigated the impact of cold storage on the lipid composition of hybrid Wagyu beef. Gas chromatography was employed to determine the fatty acid composition of the total lipids in the Loin and Round of hybrid Wagyu (Table 2). The fatty acid composition in the Loin comprised 42.5% saturated fatty acids (SFAs), 52.5% monounsaturated fatty acids (MUFAs), and 3.4% polyunsaturated fatty acids (PUFAs). In contrast, the Round exhibited 38.5% SFAs, 56.9% MUFAs, and 3.4% PUFAs. Notably, our data revealed a higher percentage of oleic acids and palmitoleic acids in the Round compared with the Loin. The overall lipid composition did not show significant changes during cold storage in either the Loin or Round (Figure S2).

Triacylglyceride (TG) composition was assessed using high-performance liquid chromatography to determine the ratio of TG molecular species (Table 3). The TG composition was similar to that previously reported [6], with the 14 major molecular species of TG accounting for >90% of the total TG composition. Regarding the ratio of each TG species, TGs (POO, OOO, and PPoO) containing MUFAs were found in higher proportions in the Round, whereas TGs (SOO and POS) containing SFAs were more prevalent in the Loin.

**Figure 5:**
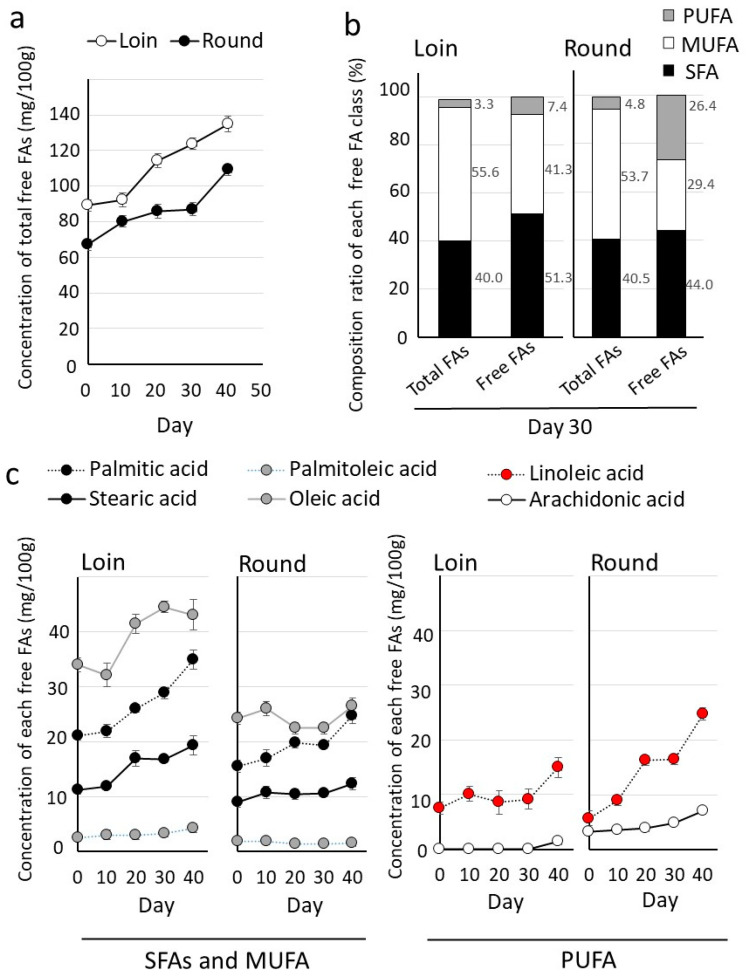
Changes in free fatty acid levels in cold storage. (**a**) Increase in total free fatty acid (FFA) levels during cold storage. (**b**) Fatty acid composition ratio of total fatty acids to FFAs on Day 20. (**c**) Increase in each FFA during cold storage. Graphs show mean values of FFAs (*n* = 5). Error bars mean ± SD.

FFAs formed through lipid hydrolysis during storage play a pivotal role in flavor development [26]. The FFA fraction was separated from the total lipids, and the FFAs were quantified using gas chromatography. The results revealed an increase in FFA levels during wet aging in both the Loin and Round (Figure 5a). Notably, the composition of the FFAs differed from that of total fatty acids, with the FFAs exhibiting a lower ratio of MUFAs and a higher ratio of SFAs and PUFAs on Day 30 (Figure 5b).

The levels of individual FFAs exhibited an increasing trend during cold storage for each fatty acid (Figure 5c). When comparing the FFA profiles of the Loin and Round, SFA and MUFA levels were higher in the Loin, whereas PUFA levels were higher in the Round. In particular, free linoleic acid content was significantly higher in the Round (16.5 ± 0.91 mg) than in the Loin (9.2 ± 1.84 mg). Measurements of the FFA compositions from cold-stored Loins and Rounds are shown Figure S3.

## 4. Discussion

Postmortem aging enhances meat tenderness and improves taste and flavor evaluation. This aging process is categorized into anaerobic wet aging and aerobic dry aging, which are distinguished by different microflora management approaches [27]. Wet aging involves packaging with a low oxygen and water vapor permeability and includes various vacuum techniques, such as conventional vacuum packaging, deep-draw-type vacuum packaging, and vacuum skin packaging [28]. Differences in meat quality between wet-aged and dry-aged beef are influenced by meat moisture content during aging [29]. However, the full extent of the changes in beef metabolites caused by the complex enzymatic and chemical reactions during aging remains unclear.

In this study, we employed conventional vacuum packaging, which facilitates the sustainable and stable transportation of livestock products. We tested the effect of wet aging on hybrid Wagyu beef during cold storage through sensory evaluation. Wet aging led to an increase in umami, richness, and continuity taste attributes in Loins and Rounds, varying with the cold storage duration (Figure 1). This significant increase in “richness” and “continuity” indicates an enhancement of “kokumi”, a newly discovered taste sensation [30]. Kokumi involves peptides from protein hydrolysates interacting with calcium-sensitive receptors in taste bud cells, influencing taste perception. Heightened kokumi synergizes with other tastes, augmenting flavor complexity and prolonging the aftertaste. Peptides and other metabolites reported to impact umami and kokumi [31] are hypothesized to contribute to the increased umami and kokumi in wet-aged hybrid Wagyu beef.

For a comprehensive metabolite analysis, we used GC/MS to analyze metabolite changes in hybrid Wagyu cattle during cold storage. Under our analytical conditions, more than 100 metabolites were detected in the marbling tissue, consisting of muscle tissue and intramuscular fat (Figure 2). In a previous study on Japanese Wagyu beef, a fairly high coefficient of variation (CV%) was observed, owing to the uneven distribution of intramuscular fat [16]. Hybrid Wagyu beef exhibited a lower CV% value and improved quantification compared with Japanese Wagyu beef due to the former’s lower crude fat content. Under our analytical conditions, approximately equal metabolite levels were detected in the Loin and Round of hybrid Wagyu cattle, with wet aging resulting in a time-dependent increases in free amino acid, sugar, and organic acid content in both the Loin and Round [32]. Tissue differences had a limited effect as several metabolites were consistently found in the Loin and Round (Figure 3). The reason for comparing Day 10 with Day 40 in this analysis is that Days 0 and 40 would not be an accurate comparison due to the wide range of relative values in metabolites.

Metabolomics analysis serves as an effective method for understanding trends/patterns, and identifying biological markers. Graphs depicting increases and decreases in several metabolites over time show possible markers for cold storage (Figure 4). Many metabolites are known to increase over days of wet aging [33,34]. In addition to the amino acids shown in the graph, the free amino acids (leucine, isoleucine, tyrosine, alanine, glycine, serine, threonine, methionine, valine, lysine, and aspartic acid) exhibited a linear increase until Day 30. The increase in these amino acids suggests that calpain, cathepsin, and other proteases degrade muscle fiber-associated proteins, leading to the tenderization of beef through wet aging [35]. High-performance liquid chromatography measurements showed a similar increase in 18 free amino acids (Figure S4). The glycolytic metabolites sorbitol 6-phosphate and glucosamine 6-phosphate were significantly decreased by wet aging. Notably, creatinine levels were substantially reduced in both the Loin and Round. During postmortem aging, mitochondrial inactivation due to cell death reduces ATP levels and accelerates glycolytic metabolite catabolism in the muscles [33,36]. Tricarboxylic acid metabolites, including citric acid, fumaric acid, and maleic acid, showed increased levels during wet aging, with these acids potentially affecting sour taste [37]. Notably, measured increases or decreases in tricarboxylic acid metabolites during wet aging may differ based on the analytical method employed [38]. In addition to metabolic activity, the red muscle ratio, glucose content in different cattle breeds, and cold storage temperature conditions may affect glycolytic metabolite degradation.

In chemically modified metabolites, acetylated lysine was detected. Acetylated lysine is a post-translationally modified lysine residue found in ryanodine receptor 2, sarcoplasmic/endoplasmic reticulum calcium ATPase 2, myosin heavy chain, myosin light chain, and troponin. This suggests that acetylated lysine may result from the protease degradation of these proteins [39]. Chemical modifications of amino acids and organic acids are also likely to influence the taste of meat. Inosinic acid, a nucleotide-related metabolite, synergistically enhances the umami flavor of meat when combined with amino acids [34]. Nucleotide-related metabolite profiles during wet aging varied between the Loin and Round (Figure S1).

**Figure 6:**
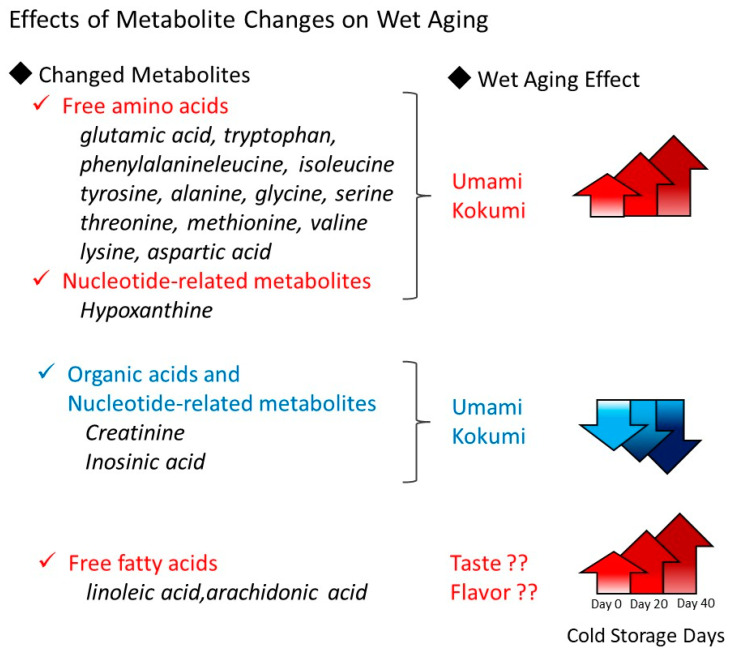
Schematic showing metabolic changes associated with wet aging. Wet aging increases free amino acid and FFA levels, depending on storage duration. Conversely, creatinine and inosinic acid are rapidly degraded. In particular, FFAs represent novel candidates for monitoring beef quality changes due to wet aging.

Hybrid Wagyu’s total fatty acid composition exhibited a higher MUFA ratio (Table 2), similar to previously reported data for Japanese Wagyu [14]. Despite being an F1 hybrid, this lipid composition similarity may be due to the Japanese-style feed management of hybrid Wagyu cattle using cattle barns [6]. Aromatic compounds result from the lipid metabolite pyrolysis derived from FFAs [40,41]. The FFA profile produced by wet aging included a significant increase in linoleic acid in both the Loin and Round (Figure 5). PUFAs, primarily stored in membrane phospholipids [14,42] rather than in TG storage, served as the source of this linoleic acid (Table 3). The TG profile showed no significant decrease during wet aging (Figure S5). The production of linoleic acid likely involves endogenous lipases activated in tissues after postmortem cooling. Lipases responsible for hydrolyzing FFAs from membrane phospholipids include phospholipase A1 and A2, which differ in terms of acyl group cleavage. Lipase genes show many isoforms, with phospholipase A2 alone presenting over 30 (e.g., cPLA2, sPLA2, and iPLA2). Elucidating the molecular mechanisms underlying FFA production during wet aging poses a major challenge. Nevertheless, several examples of aged meat products confirm that the increase in FFAs during wet aging originates from membrane phospholipids [43]. FFAs, which are known to influence taste sensitivity through fat taste receptors in the taste buds [44], may be responsible for the complex tastes of umami and kokumi resulting from wet aging. Wagyu beef is characterized by a cyclic ester lactone, which contributes to its flavor during cooking. The formation of this lactone has been proposed to involve hydroxy fatty acids derived from linoleic acid [40]. However, the effect of the elevated free linoleic acid levels on hydroxy fatty acids during wet aging remains uncertain. Future research is expected to clarify the relationship between FFAs and taste (Figure 6).

## 5. Conclusions

This study utilized metabolomics analysis to determine the effects of wet aging on hybrid Wagyu beef during cold storage. Initially, sensory evaluation confirmed that wet aging enhanced the umami and kokumi taste of hybrid Wagyu beef. GC/MS-based metabolomics analysis revealed significant changes in the levels of key taste-related metabolites, including glutamate, tryptophan, phenylalanine, acetyl-lysine, xylulose, citric acid, hypoxanthine, and creatinine. Moreover, lipid analysis indicated a significant rise in linoleic acids, precursors to aromatic compounds, owing to wet aging. This study suggests that the changes in the taste of hybrid Wagyu beef resulting from wet aging are linked to alterations in the taste-forming elements, including free amino acids, organic acids, and FFAs.

## Figures and Tables

**Table 1:** Properties of the beef packaging film.

Item	Capabilities
Physical Properties	Measured Value
Oxygen permeability	24 mL/m^2^·day·MPa
Humidity permeability	2 g/m^2^/day
Outer layer	Ethylene vinyl acetate copolymer and polyamide copolymer
Core layer	Vinylidene chloride copolymer

**Table 2:** Comparison of total lipids among tissues of hybrid Wagyu cattle. Total lipids extracted from the longissimus thoracis (Loin) and adductor muscle (Round) were analyzed by gas chromatography. The values in the table represent the mean and SD of the five animals.

Fatty Acid			% of All Fatty Acid ± SD	
Name	Abbreviation	Symbol	Loin	Round
Myristic acid	C14:0	M	2.7 ± 0.32	2.4 ± 0.30
Myristoleic acid	C14:1	Mo	0.9 ± 0.16	1.1± 0.22
Pentadecanoic acid	C15:0	Pe	0.3 ± 0.09	0.4 ± 0.10
Palmitic acid	C16:0	P	26.5 ± 1.04	25.8 ± 1.59
Palmitoleic acid	C16:1	Po	3.2 ± 0.61	4.3 ± 0.60
Margaric acid	C17:0	Ma	0.8 ± 0.21	0.7 ± 0.13
Stearic acid	C18:0	S	11.9 ± 0.83	8.9 ± 0.50
Oleic acid	C18:1	O	46.7 ± 2.13	49.1 ± 1.60
Linoleic acid	C18:2	L	2.9 ± 0.47	3.0 ± 0.53
Linolenic acid	C18:3	Al	0.2 ± 0.03	0.2 ± 0.04
Dihomo-γ-linolenic acid	C20:3	D	0.1 ± 0.02	0.1 ± 0.02
Arachidonic acid	C20:4	Ar	0.1 ± 0.02	0.2 ± 0.04

**Table 3:** Comparison of triacylglyceride (TG) molecular species among hybrid Wagyu cattle tissues. Values represent the mean and SD of three animals. Three-letter TG designations indicate fatty acid composition and do not reflect the position of the glycerol backbone. The following were estimated to be mixtures of the TGs in parentheses with close chromatographic retention times: ^a^ POO (+SLO), ^b^ POP (+PLS), ^c^ PPoO (+MOO), and ^d^ MOP (+PLP). ^e^ “Other” indicates the sum of the peak areas of unidentified TG molecular species.

Triacylglycerides		% of All TGs ± SD	
Name	Symbol	Longissimus Thoracis	Adductor Muscle
TG (C16:0/C18:1/C18:1)	POO ^a^	29.8 ± 0.3	31.8 ± 1.9
TG (C18:0/C18:1/C18:1)	SOO	9.1 ± 1.9	6.5 ± 0.5
TG (C16:0/C18:1/C16:0)	POP ^b^	8.5 ± 0.9	8.0 ± 0.9
TG (C18:1/C18:1/C18:1)	OOO	7.9 ± 1.9	9.2 ± 0.9
TG (C16:0/C18:1/C18:0)	POS	7.7 ± 0.5	5.4 ± 0.4
TG (C16:0/C16:1/C18:1)	PPoO ^c^	7.2 ± 1.9	9.0 ± 1.3
TG (C16:0/C18:2/C18:1)	PLO	5.0 ± 1.9	4.3 ± 1.5
TG (C14:0/C18:1/C16:0)	MOP ^d^	4.7 ± 0.6	4.2 ± 0.3
TG (C18:1/C18:1/C18:2)	OOL	3.9 ± 0.6	6.0 ± 0.4
TG (C18:0/C18:1/C18:0)	SOS	2.7 ± 1.0	0.9 ± 0.6
TG (C16:0/C16:0/C16:0)	PPP	2.7 ± 0.2	2.1 ± 0.3
TG (C16:0/C16:0/C18:0)	PPS	1.4 ± 0.2	1.0 ± 0.2
TG (C16:0/C18:1/C17:0)	POMa	0.8 ± 0.7	0.7 ± 0.1
TG (C16:0/C18:0/C18:0)	PSS	0.5 ± 0.4	1.2 ± 0.7
Other ^e^	-	8.1 ± 2.2	9.9 ± 0.5

## Data Availability

The data presented in this study are available on request from the corresponding author. The data are not publicly available due to patent issues.

## Supplementary Materials

The following are available online at https://www.mdpi.com/article/10.3390/metabo14020095/s1, Figure S1. Temporal changes in nucleic acid-related metabolites during cold storage. Figure S2. Temporal changes in total fatty acid composition of the Loin and Round during cold storage. Figure S3. Temporal changes in free fatty acid composition of the Loin and Round during cold storage. Figure S4. Determination of free amino acids via high-performance liquid chromatography. Figure S5. Determination of triacylglycerides via high-performance liquid chromatography.

## Abbreviations

GC/MS	Gas chromatography–mass spectrometry
PCA	Principal component analysis
SD	Standard deviation
TG	Triacylglyceride
SFA	Saturated fatty acid
MUFA	Monounsaturated fatty acids
PUFA	Polyunsaturated fatty acids
CV	Coefficient of variation
