# Supplementary material for: New Implications of Metabolites and Free Fatty Acids in Quality Control of Crossbred Wagyu Beef during Wet Aging Cold Storage
Source: Metabolites. 2024 Jan 29;14(2):95. doi: 10.3390/metabo14020095 (PMC10890485; doi:10.3390/metabo14020095)
Supplement: Supplementary file 1 [file metabolites-14-00095-s001.zip › metabolites-2800401-supplementary.pdf]

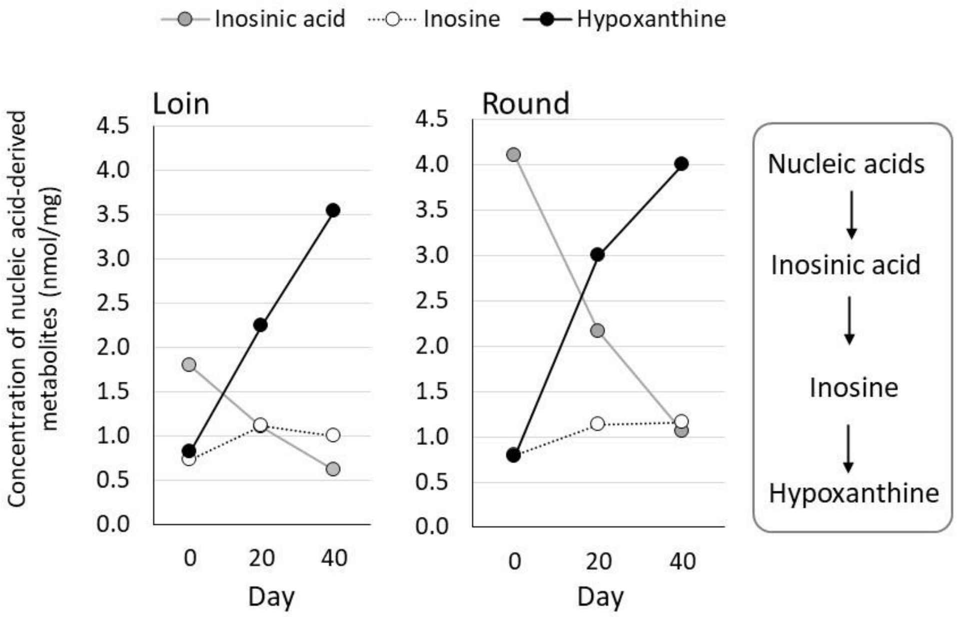

Figure S1. Temporal changes in nucleic acid-related metabolites during cold storage. Mean nucleic acid-related metabolites analyzed using high-performance liquid chromatography (n = 5). Left, longissimus thoracis (Loin); right, adductor muscle (Round). The flow diagram shows the metabolites resulting from nucleic acid degradation during postmortem aging. The conditions for high-performance liquid chromatography of Nucleic acid-related metabolites were based on the methodology described in a previous study by Ichimura et al. [24].

Loin

Name	Abbreviation	Day 0	Day 10	Day 20	Day 30	Day 40
Myristic acid	C14:0	2.7 ± 0.32	2.7 ± 0.32	2.7 ± 0.32	2.7 ± 0.31	2.3 ± 0.14
Myristoleic acid	C14:1	0.9 ± 0.16	0.9 ± 0.20	1.1 ± 0.27	1.1 ± 0.22	0.8 ± 0.11
Pentadecanoic acid	C15:0	0.3 ± 0.09	0.4 ± 0.10	0.4 ± 0.10	0.4 ± 0.10	0.4 ± 0.09
Palmitic acid	C16:0	26.5 ± 1.04	27.1 ± 1.02	25.8 ± 1.10	25.6 ± 1.22	25.4 ± 1.07
Palmitoleic acid	C16:1	3.2 ± 0.61	3.3 ± 0.70	3.3 ± 0.52	3.8 ± 0.65	3.4 ± 0.44
Margaric acid	C17:0	0.8 ± 0.21	0.8 ± 0.20	0.8 ± 0.18	0.8 ± 0.20	0.8 ± 0.17
Stearic acid	C18:0	11.9 ± 0.83	11.5 ± 0.88	10.5 ± 0.86	10.4 ± 0.78	10.9 ± 0.46
Oleic acid	C18:1	46.7 ± 2.13	46.2 ± 1.42	48.4 ± 2.36	48.4 ± 1.79	48.6 ± 1.41
Linoleic acid	C18:2	2.9 ± 0.47	3.1 ± 0.58	3.0 ± 0.49	2.9 ± 0.67	3.2 ± 0.67
Linolenic acid	C18:3	0.2 ± 0.03	0.2 ± 0.04	0.2 ± 0.04	0.2 ± 0.04	0.2 ± 0.03
Dihomo-γ-linolenic acid	C20：3	0.1 ± 0.02	0.1 ± 0.02	0.1 ± 0.02	0.1 ± 0.02	0.1 ± 0.03
Arachidonic acid	C20：4	0.1 ± 0.02	0.1 ± 0.01	0.1 ± 0.02	0.1 ± 0.01	0.2 ± 0.04

Round

Name	Abbreviation	Day 0	Day 10	Day 20	Day 30	Day 40
Myristic acid	C14:0	2.4 ± 0.30	2.3 ± 0.21	2.1 ± 0.48	2.0 ± 0.17	1.7 ± 0.10
Myristoleic acid	C14:1	1.1 ± 0.22	0.9 ± 0.15	0.5 ± 0.06	0.7 ± 0.10	0.5 ± 0.05
Pentadecanoic acid	C15:0	0.4 ± 0.10	0.5 ± 0.09	1.4 ± 0.38	0.6 ± 0.06	0.8 ± 0.15
Palmitic acid	C16:0	25.8 ± 1.59	26.4 ± 1.41	25.3 ± 1.44	25.8 ± 1.62	25.8 ± 1.21
Palmitoleic acid	C16:1	4.3 ± 0.60	3.7 ± 0.57	3.1 ± 0.59	3.2 ± 0.44	3.1 ± 0.29
Margaric acid	C17:0	0.7 ± 0.13	0.8 ± 0.15	0.8 ± 0.22	0.8 ± 0.15	0.7 ± 0.11
Stearic acid	C18:0	8.9 ± 0.50	10.0 ± 0.41	10.1 ± 0.60	10.0 ± 0.49	10.1 ± 0.23
Oleic acid	C18:1	49.1 ± 1.60	48.1 ± 1.36	47.3 ± 1.62	47.5 ± 1.12	47.5 ± 1.41
Linoleic acid	C18:2	3.0 ± 0.53	3.2 ± 0.62	3.8 ± 0.42	3.9 ± 0.56	4.8 ± 1.20
Linolenic acid	C18:3	0.2 ± 0.04	0.2 ± 0.03	0.2 ± 0.04	0.2 ± 0.03	0.2 ± 0.04
Dihomo-γ-linolenic acid	C20：3	0.1 ± 0.02	0.1 ± 0.02	0.3 ± 0.03	0.2 ± 0.01	0.3 ± 0.06
Arachidonic acid	C20：4	0.2 ± 0.04	0.2 ± 0.04	0.5 ± 0.04	0.4 ± 0.05	0.6 ± 0.08

Figure S2. Temporal changes in total fatty acid composition of Loins and Rounds with cold storage. Total lipids extracted from the longissimus thoracis (Loin) and adductor muscle (Round) were analyzed via gas chromatography. The values (%) in represent the mean and standard deviation of five cattle.

Loin

Name	Abbreviation	Day 0	Day 10	Day 20	Day 30	Day 40
Myristic acid	C14:0	3.4 ± 0.59	3.3 ± 0.66	3.7 ± 0.70	3.7 ± 0.60	3.7 ± 1.07
Myristoleic acid	C14:1	0.0 ± 0.00	0.0 ±0.00	0.4 ± 0.03	0.7 ± 0.09	0.8 ± 0.04
Pentadecanoic acid	C15:0	0.0 ± 0.00	0.0 ± 0.00	0.5 ± 0.04	0.7 ± 0.05	1.3 ± 0.06
Palmitic acid	C16:0	23.8 ± 0.92	23.8 ± 1.28	22.9 ± 0.63	23.3 ± 0.80	26 ± 1.29
Palmitoleic acid	C16:1	2.8 ± 0.52	3.3 ± 0.96	2.6 ± 0.60	2.7 ± 0.55	3.2 ± 0.62
Margaric acid	C17:0	0.0 ± 0.00	0.0 ± 0.00	0.0 ± 0.00	0.0 ± 0.00	0.4 ± 0.11
Stearic acid	C18:0	12.6 ± 0.88	13.0 ± 0.51	14.9 ± 1.25	13.6 ± 0.41	14.4 ± 1.29
Oleic acid	C18:1	38.2 ± 1.41	34.9 ± 2.31	36.4 ± 1.55	36.0 ± 0.88	32.0 ± 2.06
Linoleic acid	C18:2	8.50 ± 1.26	11.0 ± 1.51	7.50 ± 1.95	7.40 ± 1.49	11.1 ± 1.43
Linolenic acid	C18:3	0.0 ± 0.00	0.0 ± 0.00	0.0 ± 0.00	0.0 ± 0.00	0.0 ± 0.00
Dihomo-γ-linolenic acid	C20：3	0.0 ± 0.00	0.0 ± 0.00	0.0 ± 0.00	0.0 ± 0.00	0.5 ± 0.04
Arachidonic acid	C20：4	0.0 ± 0.00	0.0 ± 0.00	0.0 ± 0.00	0.0 ± 0.00	1.1 ± 0.06

Round

Name	Abbreviation	Day 0	Day 10	Day 20	Day 30	Day 40
Myristic acid	C14:0	3.8 ± 0.36	3.3 ± 0.28	2.9 ± 0.12	2.1 ± 0.24	1.6 ± 026
Myristoleic acid	C14:1	0.8 ± 0.14	0.8 ± 0.15	0.3 ± 0.04	0.5 ± 0.02	0.2 ± 0.04
Pentadecanoic acid	C15:0	0.8 ± 0.04	0.8 ± 0.17	1.4 ± 0.22	0.8 ± 0.09	0.7 ± 0.11
Palmitic acid	C16:0	23.2 ± 1.63	21.2 ± 1.83	23.0 ± 1.13	22.2 ± 0.62	22.6 ± 1.29
Palmitoleic acid	C16:1	2.8 ± 0.54	2.4 ± 0.40	1.6 ± 0.15	1.7 ± 0.26	1.4 ± 0.29
Margaric acid	C17:0	0.0 ± 0.0	0.0 ± 0.0	0.8 ± 0.05	1.0 ± 0.08	0.7 ± 0.12
Stearic acid	C18:0	13.4 ± 1.26	13.5 ± 1.40	12.1 ± 1.10	12.1 ± 0.51	11.3 ± 1.01
Oleic acid	C18:1	36.1 ± 1.70	32.4 ± 1.54	26.1 ± 1.13	25.8 ± 1.21	24.3 ± 1.23
Linoleic acid	C18:2	8.4 ± 1.99	11.1 ± 1.18	18.9 ± 1.04	18.9 ± 1.05	22.7 ± 1.07
Linolenic acid	C18:3	0.0 ± 0.00	0.0 ± 0.00	0.1 ± 0.02	0.2 ± 0.01	0.2 ± 0.02
Dihomo-γ-linolenic acid	C20：3	1.6 ± 0.27	2.2 ± 0.49	2.2 ± 0.05	2.5 ± 0.17	2.5 ± 0.19
Arachidonic acid	C20：4	4.8 ± 0.47	4.4 ± 0.28	4.4 ± 0.09	5.6 ± 0.25	6.5 ± 0.56

Figure S3. Temporal changes in the free fatty acid composition of Loins and Rounds with cold storage. Free fatty acid extracted from the longissimus thoracis (Loin) and adductor muscle (Round) were analyzed by gas chromatography. The values (%) in the table represent the mean and standard deviation of the five cattle.

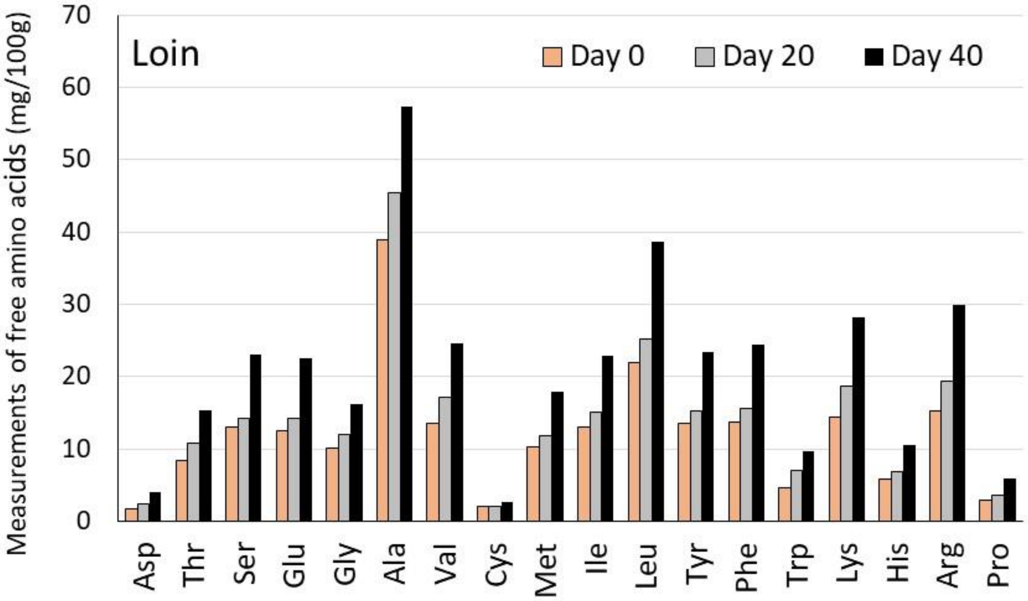

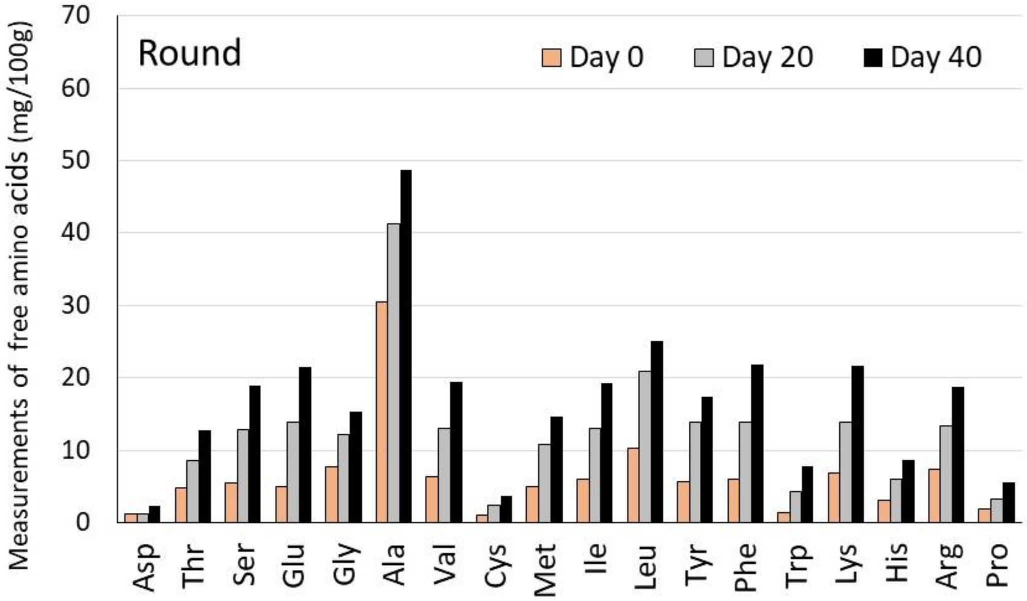

Figure S4. Determination of free amino acids by high-performance liquid chromatography. Free amino acid extracted from the longissimus thoracis (Loin) and adductor muscle (Round) were analyzed by high-performance liquid chromatography. The values in the table represent the mean of the five cattle. The conditions for high-performance liquid chromatography of free amino acids were based on the methodology described in a previous study by Ichimura et al. [24].

Loin

Name	Abbreviation	Day 0	Day 10	Day 20	Day 30	Day 40
TG (C16:0/C18:1/C18:1)	POO [a]	29.8 ± 0.32	29.4 ± 1.08	29.7 ± 0.71	29.0 ± 1.13	30.0 ± 1.45
TG (C18:0/C18:1/C18:1)	SOO	9.1 ± 1.89	9.6 ± 1.60	9.2 ± 1.41	9.1 ± 1.71	9.7 ± 0.14
TG (C16:0/C18:1/C16:0)	POP [b]	8.5 ± 0.91	8.0 ± 0.98	8.0 ± 0.63	7.6 ± 0.70	7.4 ± 0.64
TG (C18:1/C18:1/C18:1)	OOO	7.9 ± 1.91	8.9 ± 1.56	9.7 ± 1.62	9.9 ± 2.41	10.6 ± 2.11
TG (C16:0/C18:1/C18:0)	POS	7.7 ± 0.50	7.2 ± 0.19	6.5 ± 0.67	6.1 ± 0.71	6.2 ± 0.70
TG (C16:0/C16:1/C18:1)	PPoO [c]	7.2 ± 1.93	7.1 ± 1.11	7.6 ± 0.70	8.0 ± 1.09	7.3 ± 1.41
TG (C16:0/C18:2/C18:1)	PLO	5.0 ± 1.90	4.6 ± 1.61	4.4 ± 1.44	4.2 ± 1.68	4.5 ± 1.44
TG (C14:0/C18:1/C16:0)	MOP [d]	4.7 ± 0.57	4.1 ± 0.74	4.1 ± 0.36	4.8 ± 1.52	4.1 ± 0.25
TG (C18:1/C18:1/C18:2)	OOL	3.9 ± 0.59	4.3 ± 0.32	5.1 ± 0.53	5.4 ± 0.98	5.0 ± 1.13
TG (C18:0/C18:1/C18:0)	SOS	2.7 ± 0.98	2.5± 0.31	2.2 ± 0.20	2.0 ± 0.33	2.4 ± 0.44
TG (C16:0/C16:0/C16:0)	PPP	2.7 ± 0.22	2.6 ± 0.11	2.7 ± 0.48	2.4 ± 0.23	2.4 ± 0.33
TG (C16:0/C16:0/C18:0)	PPS	1.4 ± 0.21	1.4 ± 0.14	1.0 ± 0.05	0.9 ± 0.29	1.1 ± 0.11
TG (C16:0/C18:1/C17:0)	POMa	0.8 ± 0.74	1.0 ± 0.10	1.0 ± 0.15	1.2 ± 0.30	1.2 ± 0.31
TG (C16:0/C18:0/C18:0)	PSS	0.5 ± 0.45	0.6 ± 0.03	0.7 ± 0.26	0.4 ± 0.39	0.2 ± 0.31
Other [e]	-	8.1 ± 2.73	8.8 ± 1.80	8.2 ± 1.71	8.9 ± 2.84	7.8 ± 4.06

Figure S5. Temporal changes in tryacylglyceride composition in Loins with cold storage. Triacylglycerides (TGs) analyzed using high-performance liquid chromatography. The values in the table represent the mean (%) and standard deviation of three animals.
